# Molecular mechanism and clinical impact of APOBEC3B-catalyzed mutagenesis in breast cancer

**DOI:** 10.1186/s13058-014-0498-3

**Published:** 2015-01-21

**Authors:** Reuben S Harris

**Affiliations:** University of Minnesota, Department of Biochemistry, Molecular Biology and Biophysics, Masonic Cancer Center, Institute for Molecular Virology, and Center for Genome Engineering, Minneapolis, MN 55455 USA

## Abstract

Cancer genomic DNA sequences enable identification of all mutations and suggest targets for precision medicine. The identities and patterns of the mutations themselves also provide critical information for deducing the originating DNA damaging agents, causal molecular mechanisms, and thus additional therapeutic targets. A classic example is ultraviolet light, which crosslinks adjacent pyrimidines and leads to C-to-T transitions. A new example is the DNA cytosine deaminase APOBEC3B, which was identified recently as a source of DNA damage and mutagenesis in breast, head/neck, cervix, bladder, lung, ovary, and to lesser extents additional cancer types. This enzyme is normally an effector protein in the innate immune response to virus infection but upregulation in these cancer types causes elevated levels of genomic C-to-U deamination events, which manifest as C-to-T transitions and C-to-G transversions within distinct DNA trinucleotide contexts (preferentially 5’-TCA and 5’-TCG). Genomic C-to-U deamination events within the same trinucleotide contexts also lead to cytosine mutation clusters (kataegis), and may precipitate visible chromosomal aberrations such as translocations. Clinical studies indicate that APOBEC3B upregulation correlates with poorer outcomes for estrogen receptor-positive breast cancer patients, including shorter durations of disease-free survival and overall survival after surgery. APOBEC3B may therefore have both diagnostic and prognostic potential. APOBEC3B may also be a candidate for therapeutic targeting because inhibition of this non-essential enzyme is predicted to decrease tumor mutation rates and diminish the likelihood of undesirable mutation-dependent outcomes such as recurrence, metastasis, and the development of therapy resistant tumors.

## Introduction - passive versus active mutational processes in cancer

**Figure 1:**
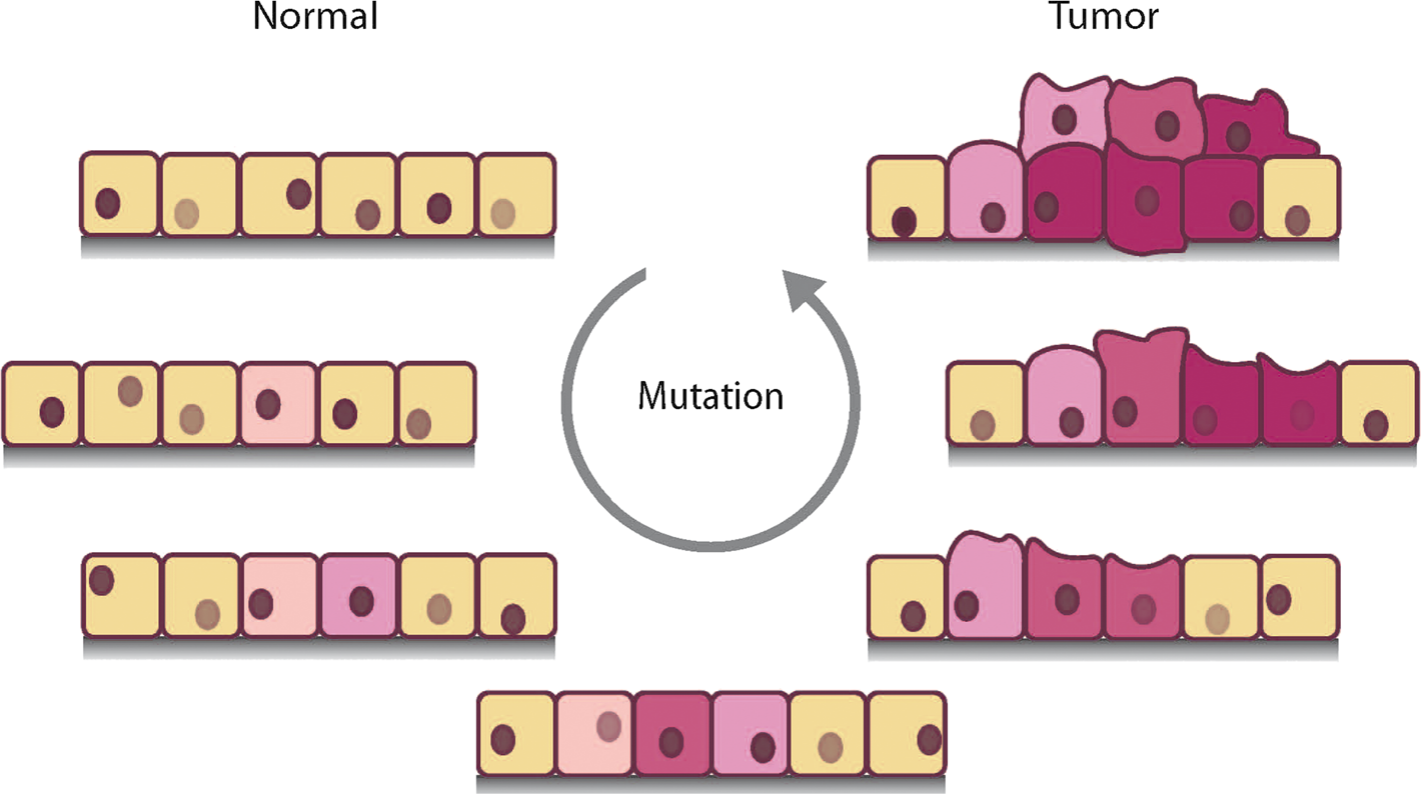
**Ongoing mutation drives cellular transformation.** A cartoon depicting the transformation of a normal cell into a mass of tumor cells. Cellular changes are represented by altered morphologies and ever-increasing shades of red, due to ongoing mutational processes represented by the inset arrow.

During all developmental stages, even when cells are not actively dividing, our DNA is subjected to continual damage by a wide variety of agents and mechanisms. The majority of these insults are mitigated by DNA repair processes, which usually restore the original DNA sequence in an error-free manner. However, some DNA damage events escape repair and manifest as somatic mutations. These mutations, by and large, occur randomly across the genome over the course of an individual’s lifetime. In some instances, however, the 'wrong combination' of somatic mutations can transform a normal cell into a tumor cell (Figure 1). The ongoing accumulation of additional mutations also contributes to the growth of local tumor cells, the development of metastatic outgrowths, and the emergence of therapy resistance.

Breast cancer is an extremely heterogeneous disease [1-3]. This review discusses the likelihood that a considerable proportion of this heterogeneity and associated phenotypes are due to a dominant acting enzyme called apolipoprotein B editing catalytic subunit 3B (APOBEC3B; see Box 1) that deaminates genomic DNA cytosines, promotes higher than normal mutation rates, and thereby enables accelerated tumor evolution. APOBEC3B does not fit into classical tumor suppressor/oncogene paradigms. Rather, it defines a new class of cancer facilitator, a type of 'enabling characteristic' [4], because it promotes the genetic diversity that provides tumors with increased adaptive capabilities. For instance, in contrast to kinase activation, which can provide a tumor cell and all of its descendants with a measurable growth advantage, APOBEC3B will cause a different repertoire of mutations in every cell it affects. APOBEC3B could have been responsible for the initial kinase activating mutation, as well as additional genetic changes that occur in descendent cells, including therapy resistance mutations, especially if they confer a selective advantage. However, APOBEC3B will not confer the same overt phenotype in every cell in which it is expressed; rather, the compounded effects of the somatic mutations will dictate the overall phenotype of each cell and the larger tumor.

Before proceeding, it is important to distinguish somatic and germline mutations. Germline mutations are inherited from our parents. These are most frequently passed from generation to generation in a Mendelian manner, but they can also arise *de novo* in our parent’s germ cells. The best-known examples in breast cancer occur in the *BRCA1* and *BRCA2* genes [5]. Mutations in *BRCA1* and *BRCA2* can compromise recombination repair, which results in elevated rates of some types of DNA damage and, consequently, an elevated risk of acquiring a cancerous combination of mutations [6]. Additional common inherited DNA repair defects are unlikely to have major roles in breast cancer, although corrupted DNA repair processes clearly contribute to many other tumor types. A prominent example is inherited defects in mismatch repair (MMR), which cause persistence of DNA replication errors, elevated mutation rates, and predisposition to colorectal tumors [7]. Moreover, *BRCA2* is also known as *FANCD1*, because some mutations in this gene predispose to a syndrome called Fanconi anemia, characterized by bone marrow failure and blood cancer [8]. In comparison, somatic mutations occur during all stages of development, including carcinogenesis. These mutations are attributable to diverse types of DNA damage that by genetic or stochastic means escape repair.

The recombination and mismatch repair processes discussed above can be considered ‘passive mutational processes’, and the DNA repair enzymes involved genomic ‘custodians’ or ‘caretakers’ [6,9]. The sum of all DNA damage events that escape repair can be considered, for lack of a better term, the spontaneous mutation level. These passively and randomly acquired mutations will happen regardless of other factors and, therefore, are largely ‘unavoidable’. The overall spontaneous mutation level and the associated composite mutation pattern are therefore expected to be similar from person to person within the human population. Defects in a particular repair process will result in an elevation of the type(s) of DNA damage typically repaired by this system.

**Figure 2:**
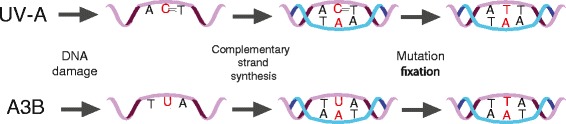
**Local mutation preferences for UV-A and APOBEC3B-induced mutagenesis.** Top row (a): ultraviolet (UV)-A crosslinks adjacent pyrimidine bases (lesion depicted by double dash sign (=)). Several DNA polymerases will bypass this lesion by inserting two adenines. Excision repair or another round of DNA replication will convert these C/A mispairs into C-to-T transition mutations. Bottom row (b): APOBEC3B (A3B) catalyzes the hydrolytic deamination of single-stranded DNA cytosine into uracil (lesion depicted in biochemically preferred 5’-TCA context). Uracil in DNA is recognized as a ‘normal’ thymine by DNA polymerases, and it therefore templates the insertion of an adenine in the complementary DNA strand. Uracil base excision repair or another round of DNA replication will convert the U-A base pair into a C-to-T transition mutation. Additional mutagenic outcomes are depicted in Figure 3.

In contrast, a growing number of DNA damage sources can be considered ‘active mutational processes’. Accordingly, these processes may be ‘avoidable’ (at least, in theory). Well-known external DNA damaging agents are ultraviolet light (UV) and tobacco compounds such as nicotine-derived nitrosamine ketone (NNK), which are associated with skin and lung cancers, respectively [10,11]. UV causes DNA pyrimidine dimers (C∧C, C∧T, T∧C, and T∧T), which are substrates for nucleotide excision repair [10]. However, a lapse in error-free repair allows pyrimidine dimers to persist and template the insertion of adenine bases during lesion by-pass DNA replication (Figure 2a). If an adenine base becomes ‘mispaired’ with a UV-linked cytosine, the next round of DNA replication or repair yields a C-to-T transition mutation in a dipyrimidine context. NNK from tobacco smoke can become activated and alkylate guanine bases (*G) [11]. These adducts are ‘read’ poorly by DNA replication polymerases, resulting in *G/A mispairs that lead to G-to-T transversion mutations. This type of tobacco-induced mutation is not known to occur within any particular DNA sequence context [12].

In addition to avoidable external sources of mutation, there are at least two significant and potentially preventable internal sources of mutation. The first comprises a group of translesion synthesis (TLS) DNA polymerases including POL κ (kappa), POL ι (iota), POL η (eta), POL ζ (zeta), and REV1, which are low fidelity enzymes that lack the capacities to proofread and perform processive DNA synthesis [13]. These enzymes are recruited to sites of DNA damage to temporarily take the place of a replicative DNA polymerase and perform lesion bypass synthesis. For example, as a specialized excision repair component POL η will usually insert the correct DNA base opposite UV-induced pyrimidine dimers [14], but other DNA POLs, such as the major replicative polymerases δ and ε, and the TLS POL κ are not adapted to this type of lesion and have a tendency to insert adenine bases that result in hallmark C-to-T transitions [15] (Figure 2a). The existence of these mechanisms can be rationalized by the fact that lesion bypass synthesis is relatively benign in comparison to a failure to complete DNA replication before cell division. Notably, multiple DNA polymerases also have important roles in developing B lymphocytes by contributing to somatic hypermutation and the overall process of antibody diversification and affinity maturation, which, interestingly, is initiated by the APOBEC-related DNA cytosine deaminase AID (activation-induced DNA cytosine deaminase) [16] (elaborated below). As enzymatic sources of mutation, DNA polymerases are theoretically inhibitable and their mutagenic contributions preventable, although the consequences of doing so may be undesirable at the organismic level because the benefits of copying DNA, bypassing DNA lesions, as well as generating adaptive immunity through antibody diversification may far outweigh the disadvantages of inhibiting these processes (even selectively or transiently).

Recent work has resulted in the discovery of APOBEC3B as an active, enzymatic source of mutation in breast cancer [17]. APOBEC3B was described originally as an antiviral enzyme, and a member of a larger family of DNA cytosine deaminases [18]. APOBEC3B uses water to catalyze the conversion of cytosine to uracil bases in single-stranded DNA. Uracil in DNA is a pre-mutagenic lesion because DNA polymerases ‘read’ it as thymine and pair it properly through two hydrogen bonds to adenine during DNA replication (Figure 2b). Thus, one of several hallmarks of APOBEC3B mutagenesis is C-to-T transition mutations within specific local DNA sequence contexts based on the intrinsic biochemical nature of the deaminase itself (elaborated below).

## Discovery and biological functions of enzyme-catalyzed DNA cytosine deamination

Humans have the potential to encode up to nine enzymes with DNA cytosine deaminase activity (APOBEC1, AID, and APOBEC3A/B/C/D/F/G/H), as well as two related proteins that have yet to elicit this activity (APOBEC2 and APOBEC4) [18]. The DNA cytosine deaminase activity of APOBEC1, AID, and several APOBEC3 enzymes was discovered originally using an *Escherichia coli*-based drug resistance assay [19,20]. Like all organisms, this bacterium has a characteristic mutation frequency, and cells expressing AID, APOBEC1, APOBEC3C, or APOBEC3G were shown to elicit 4- to 20-fold higher mutation frequencies. *E. coli* engineered to lack the uracil-specific DNA repair enzyme uracil DNA glycosylase (UDG) also have higher mutation frequencies. However, the combination of APOBEC expression and UDG deficiency caused synergistic increases in mutation frequency, in some instances >100-fold, demonstrating that these proteins catalyze C-to-U lesions in DNA and that uracil base excision repair counteracts most of the damage [19,20]. In addition, each APOBEC3 enzyme was shown to induce a distinct spectrum of drug-resistance mutations. AID preferentially caused mutations at cytosine bases preceded by purines (5’-AC and GC), APOBEC1 at cytosines preceded by thymines (5’-TC), APOBEC3C also at cytosines preceded by thymines but with a more relaxed distribution (5’-TC), and APOBEC3G uniquely at cytosines preceded by another cytosine (5’-CC). These data indicated that the DNA cytosine deamination mechanism is a conserved hallmark of this protein family and, importantly, that each enzyme has distinct intrinsic local substrate preferences. Subsequent studies have demonstrated DNA cytosine deaminase activity for all human APOBEC family members, except APOBEC2 and APOBEC4, in analogous mutator assays, biochemical studies with recombinant enzymes, and a variety of biological systems [18].

The DNA cytosine deaminase activity of AID is essential for antibody gene diversification through the distinct processes of somatic hypermutation and class switch recombination [16]. In antigen responding B lymphocytes, AID catalyzed C-to-U lesions in expressed heavy and light chain variable regions are processed by uracil base excision repair, MMR, and TLS polymerases into all six types of base substitutions. This mutagenic process is coupled to a selection process for B cells expressing antibodies with higher affinities for foreign antigen and results in large numbers of mutations within antibody gene variable regions. Simultaneously, AID catalyzed C-to-U lesions in switch regions of the expressed heavy chain gene are also processed by uracil base excision repair and MMR proteins into DNA breaks, which lead to recombination of upstream and downstream switch regions and expression of a new antibody isotype. Although these processes are complex, they are relevant here because they provide critical precedents for DNA deamination-induced mutagenesis in cancer, most importantly by demonstrating that uracil lesions can lead to both simple and complex mutational outcomes dependent upon downstream ‘repair’ processes. Moreover, considerable evidence indicates that misprocessed AID-catalyzed deamination events at immunoglobulin loci, as well as off-target deamination events, contribute to B cell lymphomas [21].

APOBEC1, the namesake, is the only family member with physiologically confirmed RNA substrates. APOBEC1 and editing cofactors are required for converting *APOB* mRNA C6666 into uracil, which yields a premature stop codon and a truncated APOB protein [22]. RNA sequencing comparisons of wild-type and *Apobec1*-null mice have revealed dozens more editing events, mostly in mRNA 3’ untranslated regions with unclear functional roles [23]. However, APOBEC1 is also a potent DNA C-to-U editing enzyme [19,24], with likely roles in innate immunity (related to APOBEC3 proteins discussed below) [18,25]. Moreover, dysregulated APOBEC1 may also play a part in cancer mutagenesis because its overexpression in transgenic mice has been shown to induce liver and colon tumors [26]. At least for mice, deep sequencing has yet to determine whether this cancer association is due to DNA and/or RNA cytosine editing activity. However, recent biochemical, computational, and functional experiments have implicated human APOBEC1 in esophageal adenocarcinomas, and tended to favor a DNA deamination mechanism [27].

Over 1,000 studies this past decade have focused on APOBEC3 enzymes because they have the remarkable capability to deaminate retrovirus and retrotransposon cDNA replication intermediates [18,25,28]. Most studies to date have focused on the mechanism of HIV-1 restriction. The current model posits that APOBEC3D/F/G/H package into assembling viral particles, travel with the particles until a new target cell is infected, and then deaminate nascent viral cDNA cytosines during reverse transcription. The resulting viral cDNA uracils then template the insertion of genomic strand adenines, and immortalize the original lesions as G-to-A mutations. Unrestrained APOBEC3 activity can completely inactivate HIV-1, but this rarely occurs *in vivo* because the viral protein Vif nucleates the formation of an E3 ubitquitin ligase complex that protects the genetic integrity of the virus by degrading the APOBEC3 enzymes. Nevertheless, APOBEC3 associated G-to-A mutations are frequently observed in viral sequences from infected patients, implying that this mutagenic process may actually facilitate HIV-1 pathogenesis by contributing to virus evolution, immune escape, and drug resistance. APOBEC3 proteins have also been implicated in restricting a large number of DNA-based parasites, including cancer-associated viruses Epstein-Barr virus, hepatitis B virus, human papilloma virus, and human T-lymphotropic virus-1 [18,25,28]. The prevailing model indicates that the entire APOBEC family provides an overlapping innate immune defense to a wide variety of known and likely many more unknown DNA-based parasites.

## Discovery and mechanism of APOBEC3B mutagenesis in breast cancer

Over the past several years, multiple lines of evidence have hinted at a novel enzymatic process in cancer mutagenesis and inspired experiments to directly test an APOBEC mutator hypothesis. First, initial speculations were provoked by the discovery of a family of enzymes with DNA deaminase activity and distinct local preferences [19,29,30]. Second, array hybridization experiments suggested upregulation of at least one APOBEC3 in several cancers [19]. Third, precedents established by AID indicated that chromosomal DNA could be susceptible to enzymatic deamination [16]. Fourth, genetic experiments in yeast indicated that APOBEC3 enzymes could mutate the genomic DNA of a eukaryote [31]. Fifth, early cancer gene DNA sequencing studies using Sanger methodology revealed mutation patterns biased to cytosine bases [32-34]. Sixth, biochemical studies showed that methyl-cytosine is more prone to spontaneous deamination than normal cytosine [35], yet in many breast tumors the majority of mutations do not occur at potentially methylated 5’-CG dinucleotide motifs [32-34]. Last, strand-coordinated cytosine mutation clusters could be induced by chemicals or APOBEC3G overexpression in yeast selection experiments [36,37], and analogous clusters biased to 5’-TC dinucleotides were discovered by deep-sequencing breast and other cancer genomes [36,38,39]. Such mutational clusters have been termed kataegis, due to a likeness with the concentration and intensity of thundershowers [38]. It is important to note that although each of these studies supports, to varying degrees, a role for enzymatic DNA cytosine deamination in cancer mutagenesis, none was demonstrative and other mechanisms were plausible [40].

The hybridization and sequencing studies cited above, plus availability of matched normal and tumor tissues and a wide variety of cell lines, indicated that breast cancer might provide an opportunity to test an APOBEC3 mutator hypothesis. Quantitative PCR assays were used to determine which APOBEC3 family members are expressed in cancerous breast tissues in comparison with matched adjacent or contralateral normal breast tissues [17]. Several family members were expressed weakly or undetectably in both tissue types (AID, APOBEC1, APOBEC2, and APOBEC4), and others were expressed similarly in both tissue types (APOBEC3A/D/F/G/H). APOBEC3C was expressed at lower levels in tumor tissues. Only one family member, APOBEC3B, was expressed at significantly higher levels in tumor tissues in comparison with matched normal tissues, and it was barely detectable in normal breast tissues [17]. Similar proportions and magnitudes of APOBEC3B overexpression were evident in RNA sequencing data sets from independent tumors [17]. Moreover, nearly two-thirds of all breast cancer cell lines showed upregulated levels of APOBEC3B in comparison with control lines such as MCF10A [17]. Taken together, these data showed that APOBEC3B is significantly and constitutively upregulated in a large proportion of breast tumors and cancer cell lines.

Two additional lines of evidence strongly linked APOBEC3B overexpression to breast cancer mutagenesis. First, elevated APOBEC3B expression levels correlated with higher C-to-T and overall base substitution mutation loads [17]. These analyses were done by comparing APOBEC3B mRNA levels and tumor mutation counts. A significant positive correlation was observed for the entire cohort, but correlations were clearest upon comparison of pooled data from the bottom and top third of APOBEC3B expressing patients. The top third has a median of 30 C-to-T mutations per exome, whereas the lower third has less than 20. Moreover, the top third has a median of nearly 70 base substitutions per exome, whereas the lower third has less than 40 (these differences are explained in a model below). Second, a series of biochemical experiments was done with the catalytic domain of APOBEC3B to deduce its intrinsic DNA cytosine deamination preference or ‘signature’ *in vitro*, and this was shown to closely resemble the actual cytosine mutation pattern in breast tumors [17]. Specifically, recombinant APOBEC3B preferred to deaminate cytosines within 5’-TCA and 5’-TCG motifs at least 5-fold and, in many instances, >50-fold better than any other trinucleotide-containing single-stranded DNA. The same two trinucleotides, 5’-TCA and 5’-TCG, were the most commonly mutated cytosine-containing motifs in two independent breast cancer mutation data sets.

Finally, genetic knockdown experiments were used to demonstrate that APOBEC3B is responsible for elevated levels of DNA damage and mutation in several breast cancer cell lines [17]. APOBEC3B mRNA knockdown caused a corresponding depletion of all measurable DNA deaminase activity in breast cancer cell line nuclear extracts. APOBEC3B knockdown cells also had lower steady state levels of genomic uracil as quantified by mass spectrometry and lower mutation frequencies as judged by drug resistance experiments. Moreover, fewer C-to-T mutations were found in the TP53 and c-MYC genes of APOBEC3B-depleted cells in comparison with controls.

Independent studies have since implicated APOBEC3B mutagenesis in other types of cancer, including head/neck, lung, bladder, cervical, ovarian, and as many as 16 out of 30 cancers examined to date [41-47]. Additional work with yeast has suggested that both the immediate product of DNA deamination, uracil, and the derivative downstream lesion, the abasic site, may be important intermediates in APOBEC3B mutagenesis [48,49]. Specifically, expression of various APOBEC3 family members in yeast induced high levels of C-to-T and C-to-G mutations, as well as kataegic clusters, and deleting uracil DNA glycosylase collapsed the mutation pattern to dispersed C-to-T mutations. Deletion of the TLS DNA polymerase REV1 as well as expression of a REV1 catalytic mutant also caused similar phenotypes. Therefore, C-to-G transversions in yeast are most likely due to REV1-dependent deoxy-cytidine insertion opposite an abasic site created by the combined action of APOBEC3B deamination and UDG base excision. Kataegis events most likely require APOBEC3B at two levels, first to create a lesion that results in the initial single- or double-stranded DNA break, and second to deaminate (perhaps processively) the resulting exposed single-stranded DNA repair intermediates. Because UDG is uniquely able to excise uracils from both double- and single-stranded DNA substrates, these clustered DNA C-to-U lesions lead to both C-to-T transitions and C-to-G transversions depending on the polymerase recruited to assist with repair. An additional feature of kataegis is a strong DNA strand bias with most cytosine mutations occurring on the same strand, likely reflecting the existence of an extensive single-stranded intermediate. Recent work in human cell lines has shown that a U/G mismatch in transfected plasmid DNA can lead to single-stranded regions susceptible to APOBEC3-mediated deamination [50].

**Figure 3:**
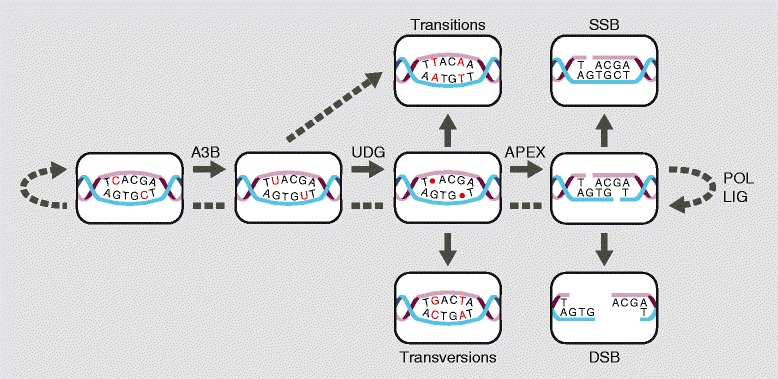
**Model for APOBEC3B mutagenesis in cancer.** The central pathway goes left to right and then circles back to depict error-free base excision repair of two C-to-U lesions catalyzed by APOBEC3B (A3B). Most genomic uracils are probably repaired in this manner. However, intermediates in this repair process can lead to a variety of mutagenic outcomes. Top left: C-to-T mutations can result from DNA synthesis over uracilated templates (as in Figure 2) or from synthesis over an abasic site because most DNA polymerases insert deoxy-adenosine opposite this non-instructional lesion. Bottom left: C-to-G transversions most likely occur when REV1 inserts deoxy-cytidine opposite an abasic site followed by repair of the original lesion or a round of DNA synthesis. Top right: a single-stranded DNA break (SSB) can result from cleavage of the phosphodiester backbone by APEX (normal component of BER). Bottom right: A double-stranded DNA break (DSB) can result from opposing APEX-mediated endonucleolytic cleavages, or from a DNA replication fork hitting a single-stranded break. Both single- and double-stranded breaks can lead to additional mutagenic outcomes such as kataegis (A3B-catalyzed deamination of exposed single-stranded DNA) and insertions, deletions, amplifications, inversions, and translocations. LIG, DNA ligase; POL, polymerase; UDG, uracil DNA glycosylase.

Overall, a compelling case has been made for APOBEC3B mutagenesis in breast and additional cancers. These studies have led to a model in which APOBEC3B-dependent DNA C-to-U deamination events underlie a variety of mutagenic outcomes (Figure 3; based on [17,41,43]). As discussed, uracils can directly template the insertion of adenines during DNA replication or local DNA synthesis. However, based on studies in yeast [48] and the fact that UDG is a very efficient enzyme that excises uracils from both single- and double-stranded DNA [51], it is likely that abasic sites are major intermediates for both dispersed and clustered C-to-T mutations and C-to-G mutations. Most polymerases will follow the ‘A-rule’ and insert an A opposite an abasic site, accounting for C-to-T transitions, whereas REV1 will uniquely insert a C opposite an abasic site, which accounts for C-to-G transversions. An additional DNA polymerase may also be involved because C-to-A mutations have been observed in other APOBEC3B-associated cancers such as ovarian carcinomas [43,45]. Uracil excision followed by abasic site processing by the major abasic site endonuclease, APEX, will generate a single-stranded break that could easily be processed into a double-stranded break by a variety of mechanisms, including the generation of a nick on the opposing strand or a replication fork collision. Nucleolytic resection of nicked or broken DNA may create single-stranded DNA substrates for APOBEC3B. As additional full genome sequences become available, it will be interesting to determine whether larger-scale chromosomal aberrations such as insertions, deletions, translocations, and amplifications correlate with APOBEC3B expression and/or define hotspots for dispersed or clustered APOBEC3B deamination events. Initial studies with breast cancer genomic DNA sequences have indicated that a portion of kataegis events may be located near sites of DNA rearrangement [36,38,52], although global levels of segmental copy number changes do not appear to correlate [44].

## Possible involvement of at least one other APOBEC family member in breast cancer mutagenesis

APOBEC3B is absent from a substantial fraction of the global population due to a 29.5 kb deletion allele that joins the homologous 3’ untranslated regions of APOBEC3A and APOBEC3B and removes the entire coding sequence [53]. The frequency of the deletion allele is high in Oceanic populations (93%), intermediate in Asian and North American Indian populations (37% and 58%), and rare in European and African populations (6% and 1%). This natural deletion allele provides clear opportunities for both molecular and clinical studies. A recent study showed that 5’-TC biased mutations and kataegis are still prevalent in a small number of breast tumors in the complete absence of APOBEC3B [54]. At first glance, this observation calls into question the aforementioned work. However, only 14 *APOBEC3B*-null breast cancers could be identified for analysis and tumor ages were unknown, making mutation frequency comparisons difficult. Therefore, the most parsimonious explanation at this time is that APOBEC3B and at least one additional family member contribute to mutagenesis in breast cancer.

The most likely candidates based on intrinsic DNA deamination preferences for 5’-TC motifs are APOBEC1 and/or APOBEC3A/C/D/F/H [18]. In particular, APOBEC3A may be dysregulated through fusion with the APOBEC3B 3’ untranslated region, poly-A region, and potential 3’ *cis*-regulatory sequences [54,55]. Consistent with this idea, several studies have shown that APOBEC3A overexpression can induce DNA damage responses resulting in the appearance of classical markers, double-stranded breaks, and elevated levels of mutation [17,56-61]. All of these studies have relied upon APOBEC3A overexpression in heterologous cell types, and it should be noted that endogenous APOBEC3A is not genotoxic even upon 100-fold upregulation by interferon-α [62,63]. Viral infections causing innate immune responses and/or splice variants may also be contributing factors. In support of this idea, recent studies on head/neck cancer have linked human papilloma virus infection to APOBEC3B upregulation, and one has shown that the viral E6 oncoprotein is sufficient to trigger APOBEC3B upregulation [46,64,65]. Hit-and-run infections causing transient APOBEC3 upregulation are also plausible but challenging to test. A current limitation is a lack of specific antibodies to many of the human APOBEC3 family members, including APOBEC3B, which if available would enable protein level studies to be compared with current mRNA-based work. In any event, additional work including clinical studies in appropriate ethnic populations will be necessary to generate larger cohorts of *APOBEC3B*-null individuals for expression studies, mutation pattern analyses, and possibly definitive functional experiments to unambiguously delineate the involvement of other family members in cancer mutagenesis.

## Clinical impact of APOBEC3B mutagenesis in breast cancer - major differences between incidence and progression

Incidence and progression are separate topics that do not necessarily have to share molecular mechanisms. Incidence is the probability of getting cancer. Progression is what happens after a cancer occurs. *A priori*, one would predict that APOBEC3B status would not impact cancer incidence because at least one upstream event is needed to elevate APOBEC3B expression [17]. However, three independent studies have indicated that homozygosity for the *APOBEC3B* null allele associates with a higher incidence of breast cancer [66-68]. The largest study showed that the risk of developing breast cancer was higher for those with homozygous null alleles than for heterozygous carriers or individuals with two copies of the intact gene [67]. Given the innate immune functions of APOBEC3B with clear antiviral and antitransposon activities [18,25,28], it is possible that *APOBEC3B*-null individuals have less protection from an as-yet-unidentified virus or they have chronically higher levels of endogenous transposition (events that would otherwise be restricted), which is another way to endow cells with a mutator phenotype. Indeed, higher levels of L1 transposition have been documented in some types of cancer [69] but association with APOBEC3B nullizygosity has yet to be addressed.

Recent work has addressed the question of progression in a large cohort of estrogen receptor-positive breast cancer patients [70]. APOBEC3B mRNA levels in previously cryopreserved samples were measured by quantitative PCR and the overall cohort average was used to divide high and low expression groups. Kaplan-Meier analyses were done to compare the two groups, and high APOBEC3B levels were found to associate with shorter durations of both disease-free survival and overall survival. Moreover, because these specimens were procured some time ago, outcomes were not complicated by adjuvant treatments. Therefore, these data indicate that APOBEC3B levels may be a pure prognostic marker for estrogen receptor-positive breast cancers. Similar results were obtained by retrospectively analyzing independent cohorts. Additional work and possibly prospective trials will be needed to evaluate estrogen receptor-negative breast cancers and deduce potential interactions with adjuvant therapies. For instance, studies will be needed to address whether elevated levels of APOBEC3B-enabled mutation contribute to the development of drug resistance and metastases.

## Conclusions

The DNA cytosine deaminase APOBEC3B is a newly defined source of DNA damage and mutation in breast cancer. It is already clear that APOBEC3B mutagenesis impacts approximately half of all breast cancers and may be responsible for large proportions of mutations in these tumors, ranging from modest to extremely high levels. Because APOBEC3B is an endogenous and likely stochastic mutagen, it is predicted to drive tumor evolution and contribute to all processes attributable to mutations (that is, tumor development, progression, heterogeneity, metastasis, and drug resistance). In fact, a recent study with head/neck cancer implicated APOBEC3B mutagenesis in activation of the kinase PIK3CA, which is also mutated in a large proportion of breast cancers [46].

To use UV light as an analogy, knowledge of its pro-mutagenic activity led to the development of measures to limit exposure and reduce the risk of potentially detrimental long-term outcomes such as skin cancer (notably sunscreen). APOBEC3B may be of similar importance to breast cancer, and one can already envisage developing the equivalent of a ‘sunscreen’ to inhibit APOBEC3B mutagenesis. APOBEC3B may be a prime therapeutic target because it is non-essential, it is rarely expressed in most normal tissues, and it is a dominant-acting enzyme with an active site that may be druggable. Proof-of-concept inhibitors have already been developed for the related enzyme APOBEC3G [71,72]. Additional work is likely to yield APOBEC3B inhibitors, and such molecules may have therapeutic merit in high-risk individuals and/or as a post-operative adjuvant to limit further tumor evolution and improve the durability and efficacy of existing therapeutics.

APOBEC3B may also have diagnostic and prognostic utility. Direct measurements of APOBEC3B mRNA or protein levels, perhaps in combination with ‘reading’ its mutagenic signature, would allow tumors to be grouped into APOBEC3B risk categories (analogous to assays for microsatellite instability in MMR-defective cancers). Such studies may determine a threshold value for APOBEC3B expression that unambiguously distinguishes a rapidly evolving cancer from an indolent mass. False positive breast cancer diagnoses still occur and molecular information on APOBEC3B may help reduce the magnitude of this problem. Appropriate diagnostic assays will also be necessary to identify cancer cases that may benefit from APOBEC3B inhibition. In conclusion, APOBEC3B mutagenesis is a major factor in breast cancer and many other human malignancies and much additional basic, translational, and clinical work is needed to fully realize this discovery.

## Box 1

APOBEC stands for apolipoprotein B editing catalytic subunit but the term ‘APOBEC’ is only appropriate for the founding member of the family, APOBEC1, which does edit *APOB* mRNA; other family members should be referred to as APOBEC-like or, ideally, by full GenBank-designated names APOBEC3A, APOBEC3B, APOBEC3C, APOBEC3D, APOBEC3F, APOBEC3G, APOBEC3H, APOBEC2, APOBEC4, and AICDA, or by commonly used alphanumeric acronyms, respectively A3A-H, A2, A4, and AID.)

## Abbreviations

AID: Activation-induced DNA cytosine deaminase
APOBEC: Apolipoprotein B editing catalytic subunit
MMR: Mismatch repair
NNK: Nicotine-derived nitrosamine ketone
POL: Polymerase
TLS: Translesion synthesis
UDG: Uracil DNA glycosylase
UV: Ultraviolet

## References

[1] Stephens PJ, Tarpey PS, Davies H, Van Loo P, Greenman C, Wedge DC, Nik-Zainal S, Martin S, Varela I, Bignell GR, Yates LR, Papaemmanuil E, Beare D, Butler A, Cheverton A, Gamble J, Hinton J, Jia M, Jayakumar A, Jones D, Latimer C, Lau KW, McLaren S, McBride DJ, Menzies A, Mudie L, Raine K, Rad R, Chapman MS, Teague J (2012). The landscape of cancer genes and mutational processes in breast cancer. Nature.

[2] Network CGA (2012). Comprehensive molecular portraits of human breast tumours. Nature.

[3] Ng CK, Pemberton HN, Reis-Filho JS (2012). Breast cancer intratumor genetic heterogeneity: causes and implications. Expert Rev Anticancer Ther.

[4] Hanahan D, Weinberg RA (2011). Hallmarks of cancer: the next generation. Cell.

[5] Couch FJ, Nathanson KL, Offit K (2014). Two decades after BRCA: setting paradigms in personalized cancer care and prevention. Science.

[6] Venkitaraman AR (2014). Cancer suppression by the chromosome custodians, BRCA1 and BRCA2. Science.

[7] Abdel-Rahman WM, Peltomaki P (2008). Lynch syndrome and related familial colorectal cancers. Crit Rev Oncogenesis.

[8] Kottemann MC, Smogorzewska A (2013). Fanconi anaemia and the repair of Watson and Crick DNA crosslinks. Nature.

[9] Vogelstein B, Papadopoulos N, Velculescu VE, Zhou S, Diaz LA, Kinzler KW (2013). Cancer genome landscapes. Science.

[10] Cleaver JE, Crowley E (2002). UV damage, DNA repair and skin carcinogenesis. Front Biosci.

[11] Hecht SS (2012). Lung carcinogenesis by tobacco smoke. Int J Cancer.

[12] Jia P, Pao W, Zhao Z (2014). Patterns and processes of somatic mutations in nine major cancers. BMC Med Genomics.

[13] Makridakis NM, Reichardt JK (2012). Translesion DNA polymerases and cancer. Front Genet.

[14] Yoon JH, Prakash L, Prakash S (2009). Highly error-free role of DNA polymerase eta in the replicative bypass of UV-induced pyrimidine dimers in mouse and human cells. Proc Natl Acad Sci U S A.

[15] Lehmann AR (2011). DNA polymerases and repair synthesis in NER in human cells. DNA Repair (Amst).

[16] Di Noia JM, Neuberger MS (2007). Molecular mechanisms of antibody somatic hypermutation. Annu Rev Biochem.

[17] Burns MB, Lackey L, Carpenter MA, Rathore A, Land AM, Leonard B, Refsland EW, Kotandeniya D, Tretyakova N, Nikas JB, Yee D, Temiz NA, Donohue DE, McDougle RM, Brown WL, Law EK, Harris RS (2013). APOBEC3B is an enzymatic source of mutation in breast cancer. Nature.

[18] Refsland EW, Harris RS (2013). The APOBEC3 family of retroelement restriction factors. Curr Top Microbiol Immunol.

[19] Harris RS, Petersen-Mahrt SK, Neuberger MS (2002). RNA editing enzyme APOBEC1 and some of its homologs can act as DNA mutators. Mol Cell.

[20] Petersen-Mahrt SK, Harris RS, Neuberger MS (2002). AID mutates E. coli suggesting a DNA deamination mechanism for antibody diversification. Nature.

[21] Robbiani DF, Nussenzweig MC (2013). Chromosome translocation, B cell lymphoma, and activation-induced cytidine deaminase. Annu Rev Pathol.

[22] Blanc V, Davidson NO (2003). C-to-U RNA editing: mechanisms leading to genetic diversity. J Biol Chem.

[23] Rosenberg BR, Hamilton CE, Mwangi MM, Dewell S, Papavasiliou FN (2011). Transcriptome-wide sequencing reveals numerous APOBEC1 mRNA-editing targets in transcript 3' UTRs. Nat Struct Mol Biol.

[24] Petersen-Mahrt SK, Neuberger MS (2003). *In vitro* deamination of cytosine to uracil in single-stranded DNA by apolipoprotein B editing complex catalytic subunit 1 (APOBEC1). J Biol Chem.

[25] Koito A, Ikeda T (2013). Intrinsic immunity against retrotransposons by APOBEC cytidine deaminases. Front Microbiol.

[26] Yamanaka S, Balestra ME, Ferrell LD, Fan J, Arnold KS, Taylor S, Taylor JM, Innerarity TL (1995). Apolipoprotein B mRNA-editing protein induces hepatocellular carcinoma and dysplasia in transgenic animals. Proc Natl Acad Sci U S A.

[27] Saraconi G, Severi F, Sala C, Mattiuz G, Conticello SG (2014). The RNA editing enzyme APOBEC1 induces somatic mutations and a compatible mutational signature is present in esophageal adenocarcinomas. Genome Biol.

[28] Malim MH, Bieniasz PD (2012). HIV restriction factors and mechanisms of evasion. Cold Spring Harb Perspectives Med.

[29] Kunkel TA, Diaz M (2002). Enzymatic cytosine deamination: friend and foe. Mol Cell.

[30] Beale RC, Petersen-Mahrt SK, Watt IN, Harris RS, Rada C, Neuberger MS (2004). Comparison of the differential context-dependence of DNA deamination by APOBEC enzymes: correlation with mutation spectra *in vivo*. J Mol Biol.

[31] Schumacher AJ, Nissley DV, Harris RS (2005). APOBEC3G hypermutates genomic DNA and inhibits Ty1 retrotransposition in yeast. Proc Natl Acad Sci U S A.

[32] Stephens P, Edkins S, Davies H, Greenman C, Cox C, Hunter C, Bignell G, Teague J, Smith R, Stevens C, O'Meara S, Parker A, Tarpey P, Avis T, Barthorpe A, Brackenbury L, Buck G, Butler A, Clements J, Cole J, Dicks E, Edwards K, Forbes S, Gorton M, Gray K, Halliday K, Harrison R, Hills K, Hinton J, Jones D (2005). A screen of the complete protein kinase gene family identifies diverse patterns of somatic mutations in human breast cancer. Nat Genet.

[33] Sjöblom T, Jones S, Wood LD, Parsons DW, Lin J, Barber TD, Mandelker D, Leary RJ, Ptak J, Silliman N, Szabo S, Buckhaults P, Farrell C, Meeh P, Markowitz SD, Willis J, Dawson D, Willson JK, Gazdar AF, Hartigan J, Wu L, Liu C, Parmigiani G, Park BH, Bachman KE, Papadopoulos N, Vogelstein B, Kinzler KW, Velculescu VE (2006). The consensus coding sequences of human breast and colorectal cancers. Science.

[34] Wood LD, Parsons DW, Jones S, Lin J, Sjöblom T, Leary RJ, Shen D, Boca SM, Barber T, Ptak J, Silliman N, Szabo S, Dezso Z, Ustyanksky V, Nikolskaya T, Nikolsky Y, Karchin R, Wilson PA, Kaminker JS, Zhang Z, Croshaw R, Willis J, Dawson D, Shipitsin M, Willson JK, Sukumar S, Polyak K, Park BH, Pethiyagoda CL, Pant PV (2007). The genomic landscapes of human breast and colorectal cancers. Science.

[35] Ehrlich M, Norris KF, Wang RY, Kuo KC, Gehrke CW (1986). DNA cytosine methylation and heat-induced deamination. Biosci Rep.

[36] Roberts SA, Sterling J, Thompson C, Harris S, Mav D, Shah R, Klimczak LJ, Kryukov GV, Malc E, Mieczkowski PA, Resnick MA, Gordenin DA (2012). Clustered mutations in yeast and in human cancers can arise from damaged long single-strand DNA regions. Mol Cell.

[37] Chan K, Sterling JF, Roberts SA, Bhagwat AS, Resnick MA, Gordenin DA (2012). Base damage within single-strand DNA underlies in vivo hypermutability induced by a ubiquitous environmental agent. PLoS Genet.

[38] Nik-Zainal S, Alexandrov LB, Wedge DC, Van Loo P, Greenman CD, Raine K, Jones D, Hinton J, Marshall J, Stebbings LA, Menzies A, Martin S, Leung K, Chen L, Leroy C, Ramakrishna M, Rance R, Lau KW, Mudie LJ, Varela I, McBride DJ, Bignell GR, Cooke SL, Shlien A, Gamble J, Whitmore I, Maddison M, Tarpey PS, Davies HR, Papaemmanuil E (2012). Mutational processes molding the genomes of 21 breast cancers. Cell.

[39] Nik-Zainal S, Van Loo P, Wedge DC, Alexandrov LB, Greenman CD, Lau KW, Raine K, Jones D, Marshall J, Ramakrishna M, Shlien A, Cooke SL, Hinton J, Menzies A, Stebbings LA, Leroy C, Jia M, Rance R, Mudie LJ, Gamble SJ, Stephens PJ, McLaren S, Tarpey PS, Papaemmanuil E, Davies HR, Varela I, McBride DJ, Bignell GR, Leung K, Butler AP (2012). The life history of 21 breast cancers. Cell.

[40] Bacolla A, Cooper DN, Vasquez KM (2014). Mechanisms of base substitution mutagenesis in cancer genomes. Genes.

[41] Burns MB, Temiz NA, Harris RS (2013). Evidence for APOBEC3B mutagenesis in multiple human cancers. Nat Genet.

[42] Lawrence MS, Stojanov P, Polak P, Kryukov GV, Cibulskis K, Sivachenko A, Carter SL, Stewart C, Mermel CH, Roberts SA, Kiezun A, Hammerman PS, McKenna A, Drier Y, Zou L, Ramos AH, Pugh TJ, Stransky N, Helman E, Kim J, Sougnez C, Ambrogio L, Nickerson E, Shefler E, Cortés ML, Auclair D, Saksena G, Voet D, Noble M, DiCara D (2013). Mutational heterogeneity in cancer and the search for new cancer-associated genes. Nature.

[43] Leonard B, Hart SN, Burns MB, Carpenter MA, Temiz NA, Rathore A, Vogel RI, Nikas JB, Law EK, Brown WL, Li Y, Zhang Y, Maurer MJ, Oberg AL, Cunningham JM, Shridhar V, Bell DA, April C, Bentley D, Bibikova M, Cheetham RK, Fan JB, Grocock R, Humphray S, Kingsbury Z, Peden J, Chien J, Swisher EM, Hartmann LC, Kalli KR (2013). APOBEC3B upregulation and genomic mutation patterns in serous ovarian carcinoma. Cancer Res.

[44] Roberts SA, Lawrence MS, Klimczak LJ, Grimm SA, Fargo D, Stojanov P, Kiezun A, Kryukov GV, Carter SL, Saksena G, Harris S, Shah RR, Resnick MA, Getz G, Gordenin DA (2013). An APOBEC cytidine deaminase mutagenesis pattern is widespread in human cancers. Nat Genet.

[45] Alexandrov LB, Nik-Zainal S, Wedge DC, Aparicio SA, Behjati S, Biankin AV, Bignell GR, Bolli N, Borg A, Børresen-Dale AL, Boyault S, Burkhardt B, Butler AP, Caldas C, Davies HR, Desmedt C, Eils R, Eyfjörd JE, Foekens JA, Greaves M, Hosoda F, Hutter B, Ilicic T, Imbeaud S, Imielinski M, Jäger N, Jones DT, Jones D, Knappskog S, Kool M (2013). Signatures of mutational processes in human cancer. Nature.

[46] Henderson S, Chakravarthy A, Su X, Boshoff C, Fenton TR (2014). APOBEC-mediated cytosine deamination links PIK3CA helical domain mutations to human papillomavirus-driven tumor development. Cell Rep.

[47] TCGA (2014). Comprehensive molecular characterization of urothelial bladder carcinoma. Nature.

[48] Taylor BJ, Nik-Zainal S, Wu YL, Stebbings LA, Raine K, Campbell PJ, Rada C, Stratton MR, Neuberger MS (2013). DNA deaminases induce break-associated mutation showers with implication of APOBEC3B and 3A in breast cancer kataegis. Elife.

[49] Chan K, Resnick MA, Gordenin DA (2013). The choice of nucleotide inserted opposite abasic sites formed within chromosomal DNA reveals the polymerase activities participating in translesion DNA synthesis. DNA Repair (Amst).

[50] Chen J, Miller BF, Furano AV (2014). Repair of naturally occurring mismatches can induce mutations in flanking DNA. Elife (Cambridge).

[51] Friedman JI, Stivers JT (2010). Detection of damaged DNA bases by DNA glycosylase enzymes. Biochemistry.

[52] Drier Y, Lawrence MS, Carter SL, Stewart C, Gabriel SB, Lander ES, Meyerson M, Beroukhim R, Getz G (2013). Somatic rearrangements across cancer reveal classes of samples with distinct patterns of DNA breakage and rearrangement-induced hypermutability. Genome Res.

[53] Kidd JM, Newman TL, Tuzun E, Kaul R, Eichler EE (2007). Population stratification of a common APOBEC gene deletion polymorphism. PLoS Genet.

[54] Nik-Zainal S, Wedge DC, Alexandrov LB, Petljak M, Butler AP, Bolli N, Davies HR, Knappskog S, Martin S, Papaemmanuil E, Ramakrishna M, Shlien A, Simonic I, Xue Y, Tyler-Smith C, Campbell PJ, Stratton MR (2014). Association of a germline copy number polymorphism of APOBEC3A and APOBEC3B with burden of putative APOBEC-dependent mutations in breast cancer. Nat Genet.

[55] Caval V, Suspene R, Shapira M, Vartanian JP, Wain-Hobson S (2014). A prevalent cancer susceptibility APOBEC3A hybrid allele bearing APOBEC3B 3'UTR enhances chromosomal DNA damage. Nat Commun.

[56] Landry S, Narvaiza I, Linfesty DC, Weitzman MD (2011). APOBEC3A can activate the DNA damage response and cause cell-cycle arrest. EMBO Rep.

[57] Suspène R, Aynaud MM, Guetard D, Henry M, Eckhoff G, Marchio A, Pineau P, Dejean A, Vartanian JP, Wain-Hobson S (2011). Somatic hypermutation of human mitochondrial and nuclear DNA by APOBEC3 cytidine deaminases, a pathway for DNA catabolism. Proc Natl Acad Sci U S A.

[58] Aynaud MM, Suspene R, Vidalain PO, Mussil B, Guetard D, Tangy F, Wain-Hobson S, Vartanian JP (2012). Human Tribbles 3 protects nuclear DNA from cytidine deamination by APOBEC3A. J Biol Chem.

[59] Carpenter MA, Li M, Rathore A, Lackey L, Law EK, Land AM, Leonard B, Shandilya SM, Bohn MF, Schiffer CA, Brown WL, Harris RS (2012). Methylcytosine and normal cytosine deamination by the foreign DNA restriction enzyme APOBEC3A. J Biol Chem.

[60] Mussil B, Suspene R, Aynaud MM, Gauvrit A, Vartanian JP, Wain-Hobson S (2013). Human APOBEC3A isoforms translocate to the nucleus and induce DNA double strand breaks leading to cell stress and death. PLoS One.

[61] Shee C, Cox BD, Gu F, Luengas EM, Joshi MC, Chiu LY, Magnan D, Halliday JA, Frisch RL, Gibson JL, Nehring RB, Do HG, Hernandez M, Li L, Herman C, Hastings P, Bates D, Harris RS, Miller KM, Rosenberg SM (2013). Engineered proteins detect spontaneous DNA breakage in human and bacterial cells. Elife.

[62] Stenglein MD, Burns MB, Li M, Lengyel J, Harris RS (2010). APOBEC3 proteins mediate the clearance of foreign DNA from human cells. Nat Struct Mol Biol.

[63] Land AM, Law EK, Carpenter MA, Lackey L, Brown WL, Harris RS (2013). Endogenous APOBEC3A DNA cytosine deaminase is cytoplasmic and nongenotoxic. J Biol Chem.

[64] Vieira VC, Leonard B, White EA, Starrett GJ, Temiz NA, Lorenz LD, Lee D, Soares MA, Lambert PF, Howley PM, Harris RS: **HPV E6 triggers upregulation of the antiviral and cancer genomic DNA deaminase APOBEC3B.** *MBio* 2014, in press. 10.1128/mBio.02234-14 PMC4278539 25538195

[65] Ohba K, Ichiyama K, Yajima M, Gemma N, Nikaido M, Wu Q, Chong P, Mori S, Yamamoto R, Wong JE, Yamamoto N (2014). In vivo and in vitro studies suggest a possible involvement of HPV infection in the early stage of breast carcinogenesis via APOBEC3B induction. PLoS One.

[66] Komatsu A, Nagasaki K, Fujimori M, Amano J, Miki Y (2008). Identification of novel deletion polymorphisms in breast cancer. Int J Oncol.

[67] Xuan D, Li G, Cai Q, Deming-Halverson S, Shrubsole MJ, Shu XO, Kelley MC, Zheng W, Long J (2013). APOBEC3 deletion polymorphism is associated with breast cancer risk among women of European ancestry. Carcinogenesis.

[68] Long J, Delahanty RJ, Li G, Gao YT, Lu W, Cai Q, Xiang YB, Li C, Ji BT, Zheng Y, Ali S, Shu XO, Zheng W (2013). A common deletion in the APOBEC3 genes and breast cancer risk. J Natl Cancer Inst.

[69] Solyom S, Ewing AD, Rahrmann EP, Doucet T, Nelson HH, Burns MB, Harris RS, Sigmon DF, Casella A, Erlanger B, Wheelan S, Upton KR, Shukla R, Faulkner GJ, Largaespada DA, Kazazian HH (2012). Extensive somatic L1 retrotransposition in colorectal tumors. Genome Res.

[70] Sieuwerts AM, Willis S, Burns MB, Look MP, Gelder ME, Schlicker A, Heideman MR, Jacobs H, Wessels L, Leyland-Jones B, Gray KP, Foekens JA, Harris RS, Martens JW: **Elevated APOBEC3B correlates with poor outcomes for estrogen receptor-positive breast cancers.** *Horm Cancer* 2014, in press. 10.1007/s12672-014-0196-8 PMC4228172 25123150

[71] Li M, Shandilya SM, Carpenter MA, Rathore A, Brown WL, Perkins AL, Harki DA, Solberg J, Hook DJ, Pandey KK, Parniak MA, Johnson JR, Krogan NJ, Somasundaran M, Ali A, Schiffer CA, Harris RS (2012). First-in-class small molecule inhibitors of the single-strand DNA cytosine deaminase APOBEC3G. ACS Chem Biol.

[72] Olson ME, Li M, Harris RS, Harki DA (2013). Small-molecule APOBEC3G DNA cytosine deaminase inhibitors based on a 4-amino-1,2,4-triazole-3-thiol scaffold. ChemMedChem.

